# Cross-species comparative analysis of single presynapses

**DOI:** 10.1038/s41598-023-40683-8

**Published:** 2023-08-24

**Authors:** Eloïse Berson, Chandresh R. Gajera, Thanaphong Phongpreecha, Amalia Perna, Syed A. Bukhari, Martin Becker, Alan L. Chang, Davide De Francesco, Camilo Espinosa, Neal G. Ravindra, Nadia Postupna, Caitlin S. Latimer, Carol A. Shively, Thomas C. Register, Suzanne Craft, Kathleen S. Montine, Edward J. Fox, C. Dirk Keene, Sean C. Bendall, Nima Aghaeepour, Thomas J. Montine

**Affiliations:** 1 Department of Pathology, Stanford University, 300 Pasteur Dr., Stanford, CA 94304 USA; 2 Department of Anesthesiology, Perioperative, and Pain Medicine, Stanford University, Stanford, CA USA; 3 Department of Laboratory Medicine & Pathology, University of Washington, Seattle, WA USA; 4 Department of Pathology/Comparative Medicine, Wake Forest School of Medicine, Winston-Salem, NC USA; 5 Department of Internal Medicine–Geriatrics, Wake Forest School of Medicine, Winston-Salem, NC USA; 6 Department of Pediatrics, Stanford University, Stanford, CA USA; 7 Department of Biomedical Data Science, Stanford University, Stanford, CA USA

**Keywords:** Machine learning, Functional clustering, Molecular neuroscience

## Abstract

Comparing brain structure across species and regions enables key functional insights. Leveraging publicly available data from a novel mass cytometry-based method, synaptometry by time of flight (SynTOF), we applied an unsupervised machine learning approach to conduct a comparative study of presynapse molecular abundance across three species and three brain regions. We used neural networks and their attractive properties to model complex relationships among high dimensional data to develop a unified, unsupervised framework for comparing the profile of more than 4.5 million single presynapses among normal human, macaque, and mouse samples. An extensive validation showed the feasibility of performing cross-species comparison using SynTOF profiling. Integrative analysis of the abundance of 20 presynaptic proteins revealed near-complete separation between primates and mice involving synaptic pruning, cellular energy, lipid metabolism, and neurotransmission. In addition, our analysis revealed a strong overlap between the presynaptic composition of human and macaque in the cerebral cortex and neostriatum. Our unique approach illuminates species- and region-specific variation in presynapse molecular composition.

## Introduction

Synapses are asymmetric intercellular junctions that differ in presynaptic neurotransmitters and postsynaptic receptors. In animals, presynaptic terminals are present in all neurons and may be the only exclusively neuronal feature among cells^1^. Despite their unique and essential role in central nervous system function^2–7^, the molecular diversity of human presynapses remains poorly understood. Indeed, a fuller appreciation of the molecular diversity of human presynapses has been obscured by technological limitations that force a trade-off in either capturing single presynapses with limited molecular information, or capturing more detailed molecular information using bulk synaptosome preparations^8–11^. Our recently developed mass cytometry- (CyTOF-) based method^12–14^, synaptometry by time of flight (SynTOF), has enabled high-throughput, multiplex analysis of single synaptic events (analogous to cellular events in CyTOF), either pre- or post-synaptic vesicles, offering a unique opportunity to characterize the molecular composition of presynaptic events at an unprecedented scale^15^.

Cross-species comparative analysis is a powerful method to understand human biological process specificity and understand biological system evolution^16^. However, integrating a large amount of heterogeneous data across multiple species requires statistically advanced tools that are computationally efficient and highly scalable. While multiple techniques are emerging to address these issues for monospecies single-event data^15,17,18^, no principled framework exists for multispecies single-event data. One strategy to bypass this is to analyze species data independently and identify single events separately^19–21^ requires identified (annotated) and well-defined single events. The limited understanding of the molecular composition of the presynapse thus precludes using this paradigm on SynTOF data.

**Figure 1:**
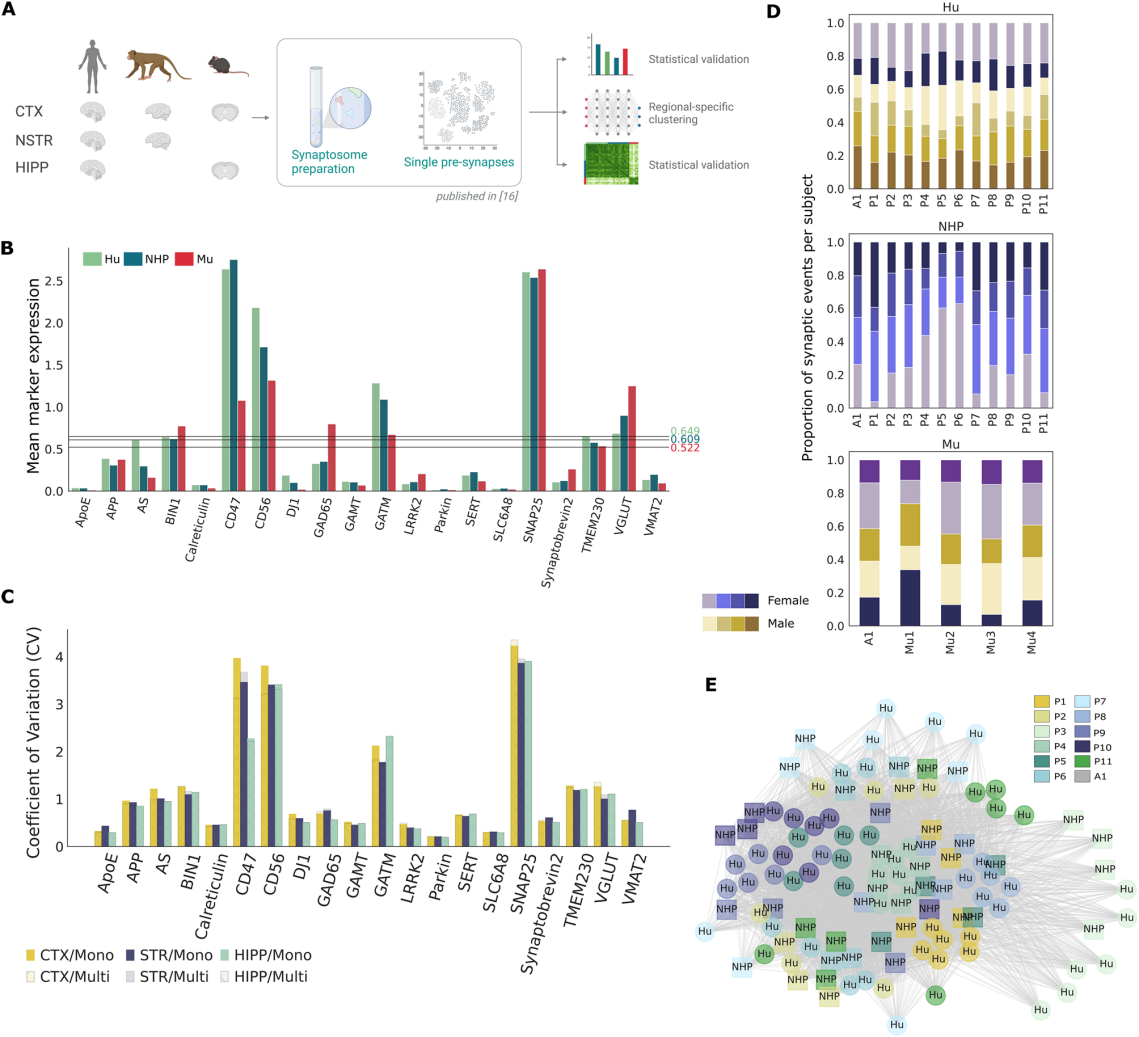
Cross-species presynaptic event comparison in cerebral cortex using a learning-based algorithm is minimally impacted by technical confounders. (**A**) Public available SynTOF data collected on synaptosome preparations from three species: human (Hu), non-human primate cynomolgus macaques (*Macaca fascicularis*, NHP), and C57Bl6 mouse (Mu) from different brain regions was leveraged in our study^17^. A region-specific machine-learning pipeline was developed to compare these more than 4 M pre-synapses between species from three different brain regions: cerebral cortex (CTX, isocortical organization) from all three species (Brodmann area 9 for Hu and frontal cortex for NHP); neostriatum (NSTR) from Hu and NHP (Mu NSTR was not collected); and hippocampus (HIPP; allocortical organization) from Hu (at the level of the lateral geniculate nucleus) and Mu (NHP HIPP was unavailable because of commitments to other projects). (**B**) Mean species cross-reactive protein expression levels for Hu, NHP and Mu in cerebral cortex. One-way ANOVA test revealed no significant differences between the three species’ mean levels (P-value > 0.05). Lines represent mean value for each species. (**C**) Coefficient of variation computed on mono-species (Hu) and multi-species dataset per protein for three brain regions. (CTX, cortex; STR, Striatum; HIPP, Hippocampus). (**D**) Proportion of subject-specific synaptic events per cluster stratified by species. Presynaptic events across samples group together based on clustering assignment, no clusters were clearly segregated by subjects or sex, suggesting that our method is unaltered by intra-species or sex variation. (**E**) Nearest-neighbor graph built using the mean expression of single events from 11 primate-specific clusters (P1–P11) and one multi-species cluster (A1) (nodes). Edges correspond to the inter and intra-species one minus the normalized Euclidean distances between two subjects. Only edges superior to the mean distance value are shown. The model derives a latent space that brings closer events from the same nature regardless of the species origin. Node positions are computed based on the Fruchterman-Reingold algorithm. (AS, alpha-synuclein).

To address this gap, we develop here an alternate, “comparative anatomy” approach that leverages an advanced machine-learning algorithm, enabling a direct cross-species comparison among the molecular composition of single presynaptic events. We leveraged publicly available single-presynapse event data: 3,657,113 from research volunteers (Hu), 759,227 from cynomolgus macaque (*Macaca fascicularis,* non-human primate NHP), and 201,261 from wild-type C57Bl/6 mouse (Mu), characterized by the expression of phenotypic antibodies^15,17,18^ related to synaptic composition and organization, and showing cross-species reactivity. This dataset was collected on research volunteers without neurologic disease or neuropathologic changes^22–25^ (n = 6, 2 females) aged 76–97 years, healthy female NHP without neuropathologic changes (n = 4, 4 females) aged 11 years, and healthy 22-month wild-type C57Bl/6 mice (n = 5, 3 females) (Fig. 1A).

Our method identifies differences and similarities between the three species in the cerebral cortex at unparalleled scale and breadth of multiplexing. It further leads to new insight into primates’ presynaptic differences in the neostriatum and Hu and Mu presynaptic divergences in the hippocampus. It reveals a strong similarity between “disease-free” (control) Hu and NHP in presynaptic molecular composition for both cerebral cortex and neostriatum, with analogous presynaptic signatures in both primates, as well as a large divergence between primates and Mu.

## Results

### Non-zero cross-reactive SynTOF markers

To address concerns about comparing interspecies data derived from antibody-based detection, we first assessed the positive mean marker expression using a one-sided t-test (Fig. S1A) and determined that our panel resulted in significant non-zero marker reactivities (P-value < 0.05). We then compared the target protein avidity of each antibody to minimize any potential impact on the observed measurements^26–31^. To do so, we compared the mean expression values using antibodies validated to react with Mu, NHP, Hu epitopes (Fig. 1B). Analysis of variance revealed no significant differences in mean expression levels of Hu, NHP, and Mu presynaptic proteins in cerebral cortex (P-value = 0.87 > 0.05 after multi-testing correction). Similarly, pairwise t-test comparisons between mean expression level of Hu and NHP presynaptic events in neostriatum (P-value = 0.93 > 0.05) and Hu and Mu presynaptic events in hippocampus (P-value = 0.99 > 0.05) also revealed no significant differences after multi-testing correction (Fig. S1B–C). These results show that the antibody panel did not have significant differences in reactivity across the three species.

In addition, no significant differences on the marker variance was observed when merging data from different species (P-value > 0.05) (Fig. 1C). Taken together, our results show that potential sources of technical variation in antibody reactivity across species are minimal and non-significant, indicating that within our defined parameters SynTOF cross-species comparison can be pursued.

### Minimal confounded model enables cross-species comparison

We explored further the extent to which species-specific variations might impact our results. To do so, our machine-learning clustering algorithm (described in “Methods”) was jointly applied to presynaptic SynTOF data from Hu, NHP, and Mu using one model per brain region, to avoid confounding our results with regional variability (Fig. 1D, E). Single events per species with a mean frequency lower than 0.01 per cluster were filtered out to abrogate the contribution of noise. Cluster consistency was validated using the silhouette score^32^.

Since assessing the correctness of a clustering method would require labeled data or prior knowledge of presynaptic composition, we assessed the impact of well-known technical confounding factors on data variations. T-distributed stochastic neighbor embedding (t-SNE) applied on the shared representation of single presynapse exhibited good mixing, without clear separation between subjects or sex (Figs. 1D, S1D). The impact of species on this clustering was evaluated by creating a nearest-neighbor graph built on the mean expression vector of each subject on each cluster, weighted using the inverse Euclidean norm (Fig. 1E). To assess the confounding of species in clustering output, only clusters gathering events from multiple species—11 clusters including both Hu and NHP (P1–P11) and one cluster gathering events from the three species (A1)—were considered when creating the graph. The observed proximity between nodes from the same clusters in the graph suggests that our algorithm created low-dimensional representation that clustered events by presynaptic event features rather than species differences^32^. A higher similarity score was observed between presynapses and marker expression within the same cluster than within each species (see Fig. S2A–D and Method).

A similar validation pipeline was applied on models trained using data from other brain regions. As a meta-analysis, we found that using a separate model for each of the three species, thus completely eliminating marker reactivity issues, resulted in the same outcome as using a single model for all species. Specifically, Hu and NHP clusters from different models retained strong correlations compared to Mu (Fig. S2E–G). In addition, no significant differences were observed between the intra- and inter-species median correlations of primate (Hu and NHP) and Mu pre-synaptic subpopulations defined either using one model for all three species (single) or separate models for each species (separate) with the same number of clusters per species (Wilcoxon’s test P-value > 0.05) (Fig. S2G).

**Figure 2:**
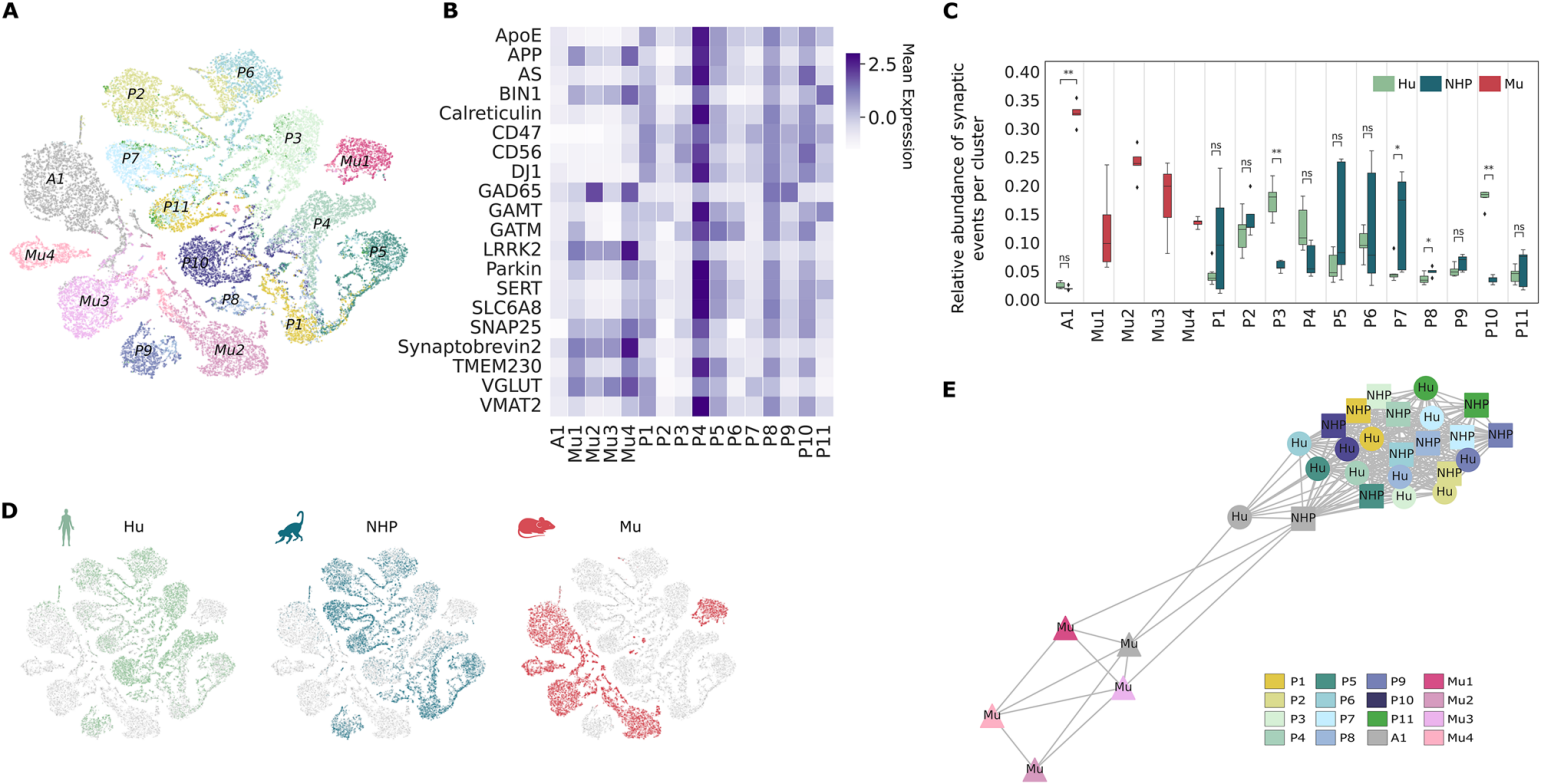
Presynaptic landscape across species in cerebral cortex. (**A**) t-SNE of single presynaptic events after nonlinear dimension reduction colored by annotated clusters. (**B**) Row-normalized cross-species mean expression heatmap of 20 proteins per cluster. (**C**) Mean frequency of presynaptic events per cluster stratified by species after removing events present in less than 0.01. Symbols indicate significant differences using Wilcoxon’s P-value < 0.05 after Benjamini–Hochberg correction. (**D**) t-SNE of single presynaptic events after nonlinear dimension reduction colored by species. (**E**) Graph based on the Fruchterman-Reingold algorithm of mean expression values displaying the underlying organization of the 15 clusters in the cerebral cortex. Nodes represent mean expression vectors embedded in the latent space, while edges indicate Pearson correlation coefficients after Bonferroni correction (P-value < 0.05). Only edges superior to the global mean Pearson correlation value were drawn. (AS, alpha-synuclein).

These results suggested the clustering method was minimally impacted by technical variability, reflecting observations using separate models (Fig. S2E–G) and the absence of technical confounder effects on our model results (Fig. 2A–C).

Finally, overlaying the original t-SNE with protein expression profiles revealed an overall high enrichment of presynaptic SNAP25 across species, spanning all clusters, with no significant differences between the three groups (P-value = 0.74 after Kruskal–Wallis test), further validating our presynaptic gating strategy (Fig. S3A, B)*.* All together, these multiple controls and checks ensure that the antibodies selected for our comparative SynTOF study yielded robust single presynapse data across species with minimal confounding.

### Distinct cerebral cortical presynaptic molecular composition between primates and Mu

The generated clusters mainly exhibited species-specific presynaptic subgroups, identifying 11 clusters exclusively with primate presynaptic events (P1–11) and 4 clusters composed entirely of Mu samples (Mu1-4) (Fig. 2A–D). Although one cluster grouped together events from all species (A1), the overall low expression for the 20 proteins observed in the A1 cluster suggests that presynaptic events present in this cluster remained largely undistinguished by the chosen antibody panel (Fig. 2B). A higher proportion of Mu events was observed compared to primate events in this cluster (Fig. 2C, D). Interestingly, VGLUT and GAD65 were found to co-expressed in one Mu-specific cluster (Mu4) and one Primate-specific cluster (P8) (Figs. 2B, S3C). Similar to^17^, one “high-expressed” primate-specific cluster was found (P4) with high expression of most of the markers (Fig. 2B) including co-expression of VGLUT, VMAT2 and SERT (Figs. 2B, S3C). Hu and NHP events were unequally distributed between primates-specific clusters, with a significant difference in event abundance between species observed in 4 clusters (P3, P7, P8, P10) (Fig. 2C). Additional statistical analysis showed high expression of GAD65 in 4 clusters (Mu2, Mu4, P8 and P9) and high expression of VGLUT in all Mu-specific clusters and 5 primate clusters (P1, P4, P5, P7 and P8) (Figs. 2B, S3B–D).

A more meaningful description of the underlying organization of the different clusters and the relative differences between species was obtained by building a Pearson correlation graph from the mean expression vectors of each species, illustrated in Fig. 2E. This graph exhibited significantly stronger correlations within primate-specific clusters than between primates and Mu, splitting the presynaptic events into two subgroups, with the multi-species cluster (A1) of unidentified events lying at the intersection of this binary partition.

This underlying molecular composition was consistent with the evolutionary tree and can be appreciated at different scales. At a high level, a cross-species correlation analysis showed lower correlation coefficients between mean expression of Mu and Hu protein levels than NHP and Hu protein levels, highlighting the close presynaptic molecular proximity between NHP and Hu compared to Hu and Mu (Figs. 2E, S3E). The resulting partitioning is also noticed at the single presynaptic event level with the t-SNE plot, depicting a species-dependent structure of the presynaptic molecular events with a large overlay between Hu and NHP samples (Fig. 2D).

The divergent nature of Hu and Mu presynaptic events in the cerebral cortex was also observed in hippocampus. Using the same pipeline, a new model was trained on presynaptic events from Hu and Mu hippocampi, generating a hippocampal low dimensional space with a few overlaps between events from the two species, corroborating our findings from the cerebral cortex (Fig. S4). Notably, three out of fourteen clusters contained events from both Hu and Mu, including two clusters of low-expressed markers and one cluster with a significantly greater number of Mu events found in the hippocampus (Fig. S4A–D). While these three Hu-Mu clusters showed high correlation, significantly higher expression of GAD65, Synaptobrevin and lower expression of DJ1 and ApoE in Mu compared with Hu distinguished the two species (Adjusted Wilcoxon’s P-value < 0.05) (Fig. S4E, F). Furthermore, hippocampal Hu-specific clusters showed similar profiles as primate-specific presynapses in the cerebral cortex: one “high expressed” human-specific cluster was generated aligning with our observation in the cerebral cortex and previous study^17^, and one Mu-specific cluster with co-expression of VGLUT and GAD65 (Fig. S4G).

**Figure 3:**
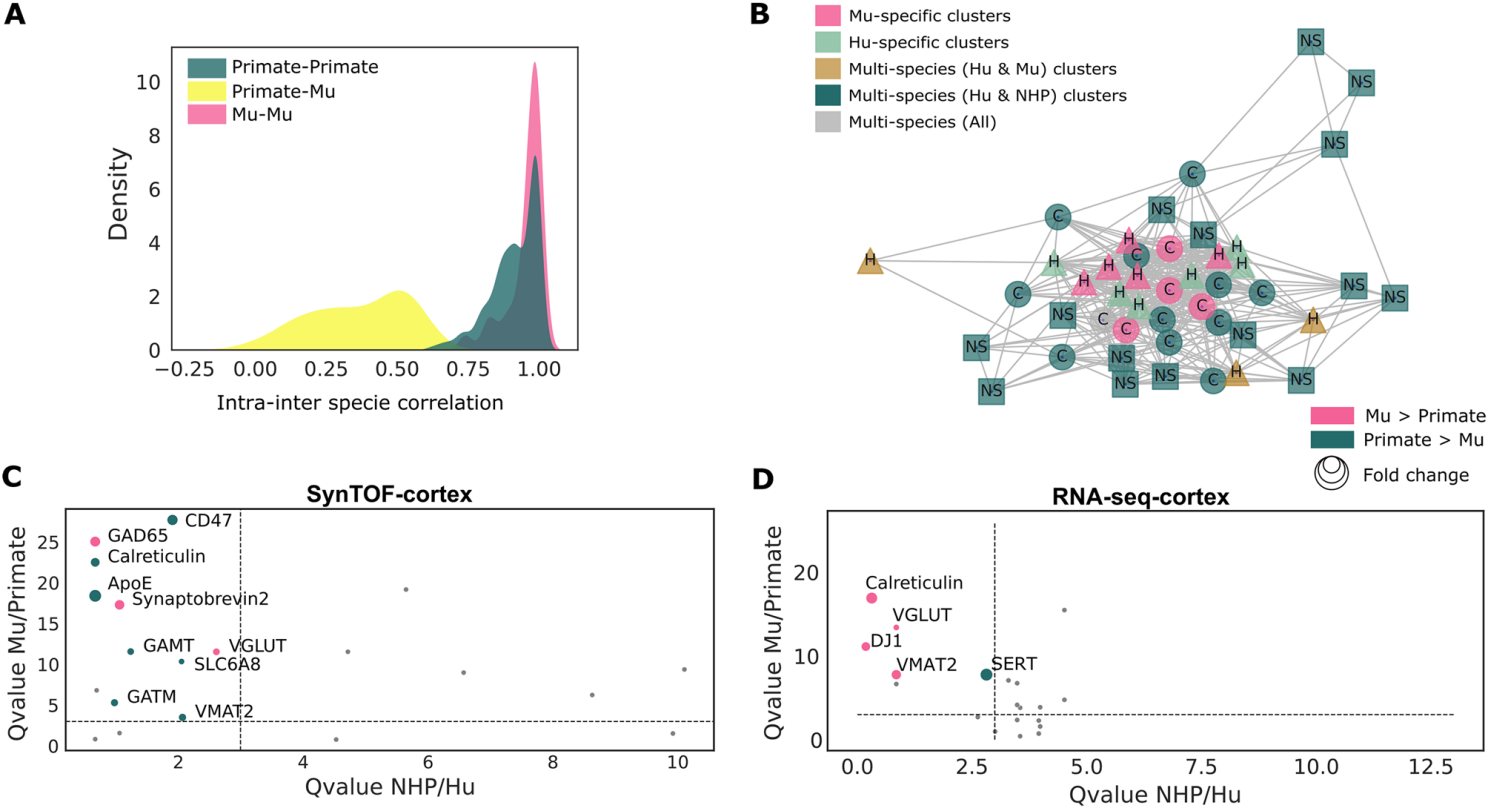
Divergence in presynaptic molecular signatures between primate and Mu in the cerebral cortex. (**A**) Inter-individual variations: density plot showing inter-species Pearson correlation coefficient distribution of markers in the cerebral cortex. (**B**) Meta-analysis across brain regions visualized through a Pearson correlation graph. Each node represents the mean expression in one brain region-dependent cluster. Edges correspond to the Pearson correlation coefficient between these nodes. Only significant edges (P-value < 0.05 using non-correlation testing corrected using Bonferroni's method), above the global mean edge value, are drawn. Nodes are colored by species-specificity of each cluster and positioned based on the Fruchterman-Reingold algorithm. (**C**) Significant pseudo-bulk presynaptic differential protein mean expression analysis between primates and Mu in cerebral cortex in SynTOF and RNA-seq data. Only significantly different proteins between Mu and primates, with fold-change greater than 0.5, and that show no significant differences between the two primates, are colored. P-values are derived using Wilcoxon's test after Benjamini–Hochberg correction. (**D**) Significant pseudo-bulk presynaptic differential protein mean expression analysis between primates and Mu in cerebral cortex in RNA-seq data from^33^. Similar to (**C**), only significantly different genes between Mu and primates that showed no differences in primates are colored. (AS, alpha-synuclein).

More broadly, inter-individual correlations were more consistent within primates or Mu than across these species (Fig. 3A). For meta-analysis across brain regions, a correlation network associating mean expression per cluster per brain region was built, visualizing a comprehensive higher correlation within Mu-specific clusters regardless of brain region, (as suggested by the proximity of this community on the graph), than across clusters including primate samples (Fig. 3B). Together this comparative analysis describes an overall quantitatively divergent nature of presynaptic molecular composition between primates and Mu.

### Pseudo-bulk analysis shows species-specific molecular profiles and weak connection between nuclear transcriptomic and presynaptic proteomic data in cerebral cortex

As no common presynaptic clusters were identified between Mu and primates, we performed a pseudo-bulk differential analysis to identify species-specific expression. To do so, the adjusted Wilcoxon test was applied on pseudo-bulk marker mean expressions between Hu and NHP cerebral cortex, and between Primate and Mu. A holistic comparative analysis of the cerebral cortex indicated significantly higher enrichment of CD47, ApoE, calreticulin, GAMT, SLC6A8, GATM and VMAT2 (P-value < 0.05), and lower expression of Synaptobrevin 2 (P-value < 1e−7), in primates compared with Mu, with no significant differences between Hu and NHP (P-value > 0.05) (Fig. 3C). Furthermore, significantly higher levels of pseudo-bulk mean expression of both GAD65, an enzyme specifically expressed by inhibitory neurons, and VGLUT, a vesicular transporter expressed by excitatory neurons, were found in Mu than in the two primates (Kruskal–Wallis test P-value < 1e−2) (Fig. S3A). Similar observations were found in the hippocampus: ApoE, calreticulin, CD47, CD56 and DJ1, had significantly higher expression in Hu, while synaptobrevin2, GAD65, VGLUT, as well as APP, TMEM and LRRK2 had significantly lower expression in Hu than in Mu (Fig. S4H).

We hypothesized that the synaptic proteomic signature across species also might be observed at the transcriptomic level by investigating the relative differences between protein abundance at the presynapse level and gene expression at the nuclear level. Exploiting publicly available transcriptomic data from Hu (n = 2), NHP (in this case marmoset) (n = 2), and Mu (n = 12)^33^, a pseudo-bulk comparison was applied to the 20 transcripts that encode the proteins targeted by our SynTOF panel. Although limited by the small cohort size, most of our presynaptic protein expression data correlated poorly with the nuclear abundance of the corresponding transcript in the motor cortex (Fig. 3D). Interestingly, the significant relative overabundance of VGLUT in Hu was conserved at both the nuclear transcript and synaptic protein levels. Although technical confounders including the cross-species brain size and relative age differences might limit this study, these results align with what has been observed by others in bulk tissue^34–36^, and emphasize the value of SynTOF in discovering the molecular composition of synapses.

### Integrated analysis between Hu and NHP presynaptic events in cerebral cortex and neostriatum exhibited strong proximity of primate presynaptic organization

**Figure 4:**
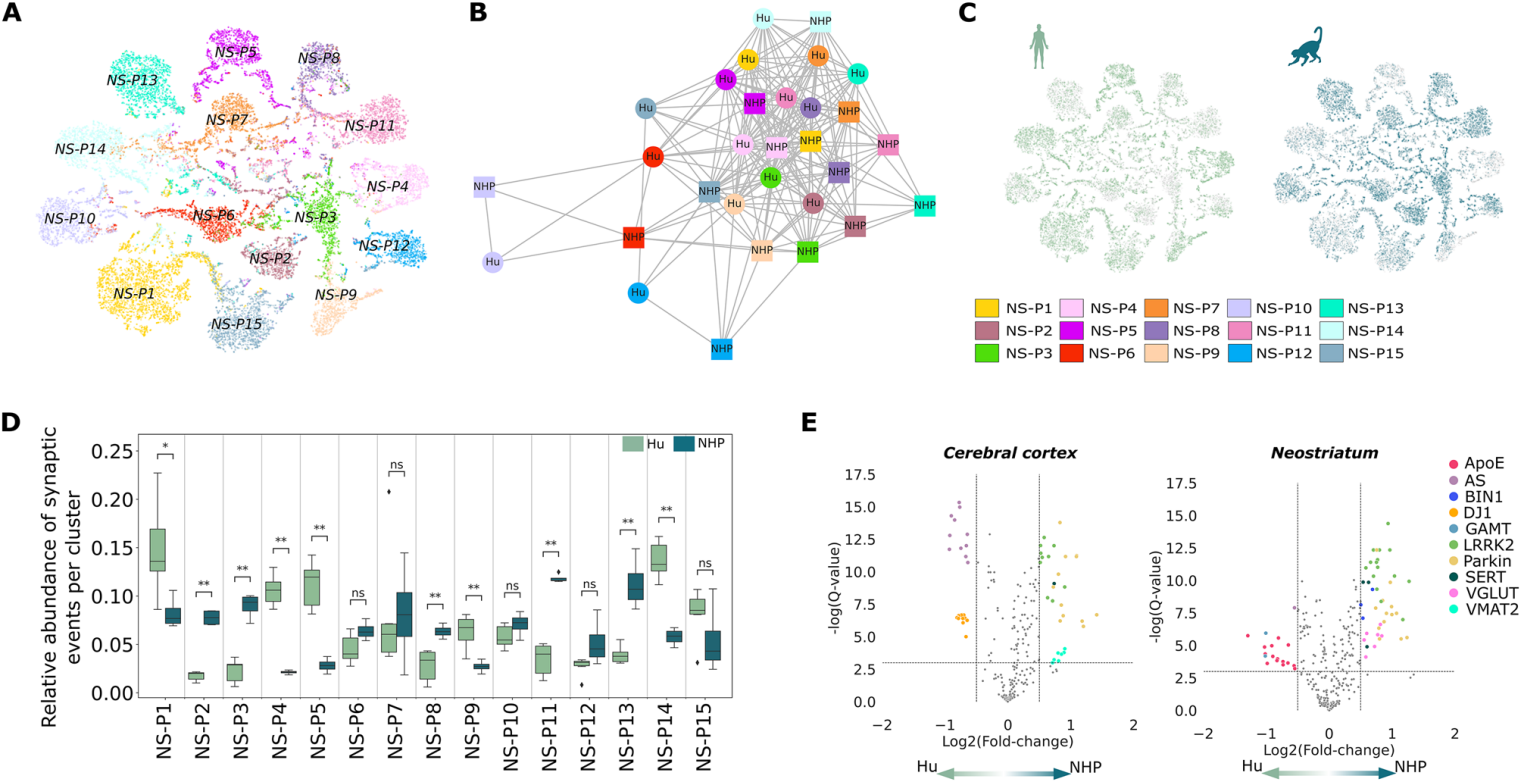
SynTOF analysis reveals a strong proximity between Hu and NHP presynaptic events with species specificity at the protein level. (**A**) t-SNE of presynaptic events after nonlinear dimension reduction colored by clusters. (**B**) Visualization of the connection between mean expression of Hu and NHP samples from different clusters. Nodes represent mean expression vectors per cluster stratified by species, edges correspond to Pearson correlation between nodes vectors. Only significant edges (P-value < 0.05 using non-correlation testing) above the global mean edge value are displayed. Homogeneity was observed across the two datasets, suggesting a high similarity between identified presynaptic events in the two species. Node positions were computed based on the Fruchterman-Reingold algorithm. (**C**) Original single events from neostriatum embedded and projected in two dimensions using t-SNE, colored by species. (**D**) Mean frequency of Hu and NHP synaptic events per cluster after removing events present in less than 0.01. Symbols indicate significant differences using Wilcoxon’s P-value < 0.05 (*) after Benjamini–Hochberg correction. (**E**) Volcano plot of differential protein expression between Hu and NHP, per cluster in cerebral cortex and neostriatum after multiple testing corrected Wilcoxon’s test. Only significantly different marker expressions are colored.

Finally, we supplemented our results from the frontal cortex by performing the same clustering analysis using NHP and Hu samples from the neostriatum (Figs. 4A, S5A). Precisely, the same unsupervised workflow (with a newly trained model) was applied on single-presynapses acquired from the neostriatum of both primates (see “Methods”). A correlation network built from species-specific mean expression per clusters brought out a stronger correlation among intra-cluster presynaptic events from different species as indicated by proximity of these vectors in the correlation network, emphasizing the overall strong similarity between primate presynaptic molecular composition for the 20 proteins analyzed (Fig. 4B). Just as for the frontal cortex, the presynaptic events from the neostriatum of the two primates blend well together, forming 15 new cross-species clusters (NS-P1-15) (Fig. 4A–C), including one “high-expressed” cluster (NS-P9) and one VGLUT-VMAT2-SERT co-expressed cluster (Fig. S5A, B). However, the relative presynaptic proportion per cluster contrasts significantly between the two species in 10 clusters (Fig. 4D).

To gain further insight of the differences between Hu and NHP, we investigated markers differentially expressed between Hu and NHP within each cluster (Fig. 4E). Using 20 phenotypic markers, we compared mean marker intensity between Hu and NHP using adjusted Wilcoxon’s test. Differences between the two primates included a relatively higher expression of DJ1 and AS in frontal cortex, and ApoE in neostriatum for Hu compared to NHP with an adjusted P-value < 0.05. Contrarily, parkin and LRRK2 in both regions, VMAT in frontal cortex and VGLUT in neostratium showed overall higher expression in NHP compared to Hu samples (FDR after correction P-value < 0.05). Minor differences in the Hu neostriatum include BIN1 and SERT (3 clusters) decreased protein expression, and AS and GAMT (2 clusters) increased expression compared to NHP (Figs. 4E, S5C).

## Discussion

Cross-species comparison of the central nervous system has a long tradition of illuminating human-specific structures and providing insight into function. Indeed, Bjornson-Hooper, et al. recently reported an extensive cross-species comparison of Hu, NHP, and Mu CyTOF data from immune cells in blood, demonstrating the importance of understanding differences between human and model organisms^37^. Here, we report our cross-species comparison of presynaptic molecular composition using publicly available SynTOF data. As far as we are aware, our study provides the first unsupervised integrating cross-species comparison of multiplexed, single presynaptic data. Several antibodies were used to gate for presynaptic events versus other debris in the homogenate, yielding 20-plex quantitative data on over 4.5 million single, highly enriched presynaptic events from the three species. These unparalleled broad, deep, and specific multispecies molecular data formed the basis of our comparative presynaptic investigation. Leveraging recent machine-learning advances, we developed the first integrated framework to compare the large cross-species datasets generated by SynTOF and investigated key differences and similarities among species and brain regions in presynaptic protein expression from disease-free (control) Hu, NHP, and Mu brain.

Analysis across multiple species can be challenging as data obtained from different groups might be confounded by unidentified technical and biological factors^26^. While many techniques have been developed to correct known unwanted variations^26–29,31^, determining which biological factors play the most important roles in cross-species investigations remains an open question. While merged cross-species data enables direct comparison, it has some potential limitations, including notably the possible technical differences across species. This potential limitation, inherent to antibody-based experiments, was addressed by conducting statistical analyses on SynTOF signals across species and computing the dispersion between single-species and multi-species datasets. These analyses revealed no significant differences in both cases (P-value > 0.05). Additional validation analysis suggested that the clustering method was minimally impacted by technical variability, reflecting observations using separate models (Fig. S2E–G) and the absence of technical confounders on our model results (Fig. 1D–F). All together, these results confirmed the minimal influence of technical variation on SynTOF signals, and allowed the comparison of synaptic subpopulations using our multi-species integrating approach despite known differences in target sequence.

We focused primarily on isocortical samples because this region was available from all three species. Our clustering analysis revealed two non-overlapping types of presynapse clusters: primate-specific and Mu-specific; pseudo-bulk comparative analysis of primate and Mu protein expression in cerebral cortex exhibited 8 significantly higher protein levels in primates including components of “eat-me, don’t eat me” signaling between synapses and microglia (ApoE, calreticulin, and CD47)^38^, creatine metabolism (GAMT, GATM and SLC6A8), and neurotransmission (VMAT2)^39–42^. In contrast, 3 proteins involved in the machinery of neurotransmission (GAD65, synaptobrevin2, VGLUT) had a significantly lower expression in primates. A low correlation was found between presynaptic protein expression and the transcriptomic abundance of these proteins in the motor cortex^34,43,44^ with the exception of lower expression of VGLUT at both the presynaptic protein and nuclear transcriptomic levels (Fig. 3C).

We supported our analysis in cerebral cortex by comparing SynTOF data from Hu and Mu in the allocortical hippocampus. The clustering algorithm revealed a small overlap between single presynapses from the two species, corroborating the pervasive difference in the molecular composition of presynapses in Hu and Mu. Although most of our results aligned well with existing knowledge of the molecular composition of synapses, some synaptic profiles were unexpected, such as GAD65 + VGLUT + SERT in H-Mu4 (Fig. S4C), and should be interpreted cautiously until an alternative highly multiplexed single-synapse technology is available that can validate our results. Pseudo-bulk analysis performed on hippocampus data and led to similar significant differences between Hu and Mu presynaptic protein levels, which overlapped with isocortical presynapses (*i.e*., increased ApoE, calreticulin, CD47 and decreased VGLUT, synaptobrevin-2, GAD65 in Hu compared to Mu). These data support significant differences in both isocortical and hippocampal presynapse protein abundance important to basic functions such as neurotransmission, energy metabolism, and synaptic pruning.

Although differences in protein expression were observed between NHP and Hu, the generated clusters did not identify any Hu-only or NHP-only subgroups, indicating strong proximity between the molecular composition of presynapses for the two primates in both frontal cortex and neostriatum. In the frontal cortex, where only 6 presynaptic proteins expressed significantly different between Hu and NHP across all clusters AS and DJ1 expression was higher in Hu, while LRRK2 and parkin showed lower expression in Hu. In neostriatum, the significant cross-cluster differences between Hu and NHP presynaptic protein levels validated higher expression of ApoE in Hu and lower expression of the same three proteins (LRRK2, Parkin) in Hu.

There are several limitations to our study, including those inherent in using available data^15^ that, by nature, will in hindsight have missed opportunities for maximum utility. First, our study is of course limited by the 20 marker panel chosen to characterize the presynaptic particles across species. Specifically, around 32% of Mu pre-synapses and less than 3% of primate pre-synapses are undistinguished by our panel (Fig. 2C). In addition, a practical limitation in this study was the enormous difference in the size of the cerebral cortex between the three species. We used a region of prefrontal cerebral cortex (Brodmann area 9) from Hu, prefrontal cortex from NHP, and all of cerebral cortex from Mu. In theory, this variation in subregion of cerebral cortex in the different species and differences in potential projections from synapsing cells originating in other region might confound our results; Similarly, cellular composition and distribution^34,45^, synapse density^46^, network organization^47,48^, morphological features^49^ can vary across species. These variations could potentially contribute to the observed differences at the presynaptic level. Indeed, Bakken et al., reported a broadly conserved cellular taxonomy across mice, marmosets and humans albeit with differences in cellular proportion and gene expression in the motor cortex. Furthermore, they found a larger overlap of neuronal cell type composition between humans and marmosets (39%) than primates and mice (27%) along with a significant difference in the ratio of excitatory to inhibitory neurons (2:1 in humans, 3:1 in marmosets, and 5:1 in mice). These species-specific cellular profiles may explain the substantial overlap observed between Hu and NHPs presynapses, as well as the weaker similarity between primates and mice presynaptic composition. However, we expect that it has limited impact because we already have shown no significant difference in data from this same SynTOF panel from Hu temporal versus parietal cortex^17^.

Age also was a potential source of variability in our study. Approximated as percent of maximum lifespan, the humans had lived 73% ± 4.8, the macaques had lived 40%  ± 0.9, and the mice had lived 96% of their lifespan. While Hu and Mu were comparably aged relative to maximum lifespan, NHP were relatively younger. Differences in age may influence the observed pre-synaptic composition, given the diverse lifetime of synaptic proteins. Recently^50^, revealed the heterogeneity and wide range of synapse protein lifetimes in various regions of the mouse brain. Although the lifespan and diversity of synaptic proteins in primates remain unexplored, synapse density and function were found altered during brain aging^51,52^. However, we found many differences in presynapse protein abundance between the similarly aged Hu and Mu, suggesting that these are valid species differences relatively uncompromised by effects of aging. We observed many fewer regional differences in SynTOF presynaptic signal between Hu and NHP, suggesting that at least for these 20 proteins there may be limited impact of aging when both clinical and pathologic examinations are used to exclude subclinical “age-related” diseases. A more comprehensive understanding of synapse protein lifetime across species would enhance our understanding of synaptic function and diversity.

In summary, we proposed a machine learning framework to compare presynapse molecular abundance across three species and three brain regions. After extensive analysis to assure the validity of cross-species comparison of SynTOF data, we observed significant differences in protein abundance of primate (Hu and NHP) vs. Mu presynapses with respect to synaptic pruning, cellular energy, lipid metabolism, and neurotransmission. In contrast, there was strong overlap between presynaptic molecular composition of Hu and NHP presynapses in both cerebral cortex and neostriatum. As expected, presynaptic composition correlated with evolutionary distance. More divergent presynaptic landscapes were observed between Hu and Mu (~ 87 MYA) than between Hu and NHP (~ 28.9 MYA)^53^, aligning with cross-species transcriptome and epigenome comparative analysis^33^.

Brain species specificity has been extensively studied at the cell level, comparing transcriptome expression from various brain regions. Our proteomic results did not correlate well with nuclear transcriptome, as has been observed by others in bulk tissue, and thereby provide a unique perspective on the comparative molecular composition of presynapses that may guide functional insight in humans and other species.

## Methods

All SynTOF data used in the present study were generated in a previous publication^17^ and are publicly available. Please, see the Supplemental Methods for details. As described therein human, macaque, and mouse synaptosomes were prepared using established protocols^10^, modified for CyTOF analysis^18^, including mass tag barcoding^15^.

### Unsupervised deep-learning approach for interspecies clustering

Identification of subpopulation similarities and differences between species usually relies on an integrated clustering assignment pipeline^54–57^. However, clustering in high-dimensional space is challenging, due to the unreliability of similarity metrics^58^. Dimension reduction techniques, such as Principal Component Analysis (PCA), circumvent this issue by lowering the dimensionality of the input data. Yet, the representative power of popular dimension reduction algorithms such as PCA is limited due to assumptions made about the data (e.g., linearity).

To bypass this issue, we leverage neural networks and their attractive property to model complex relationships between high dimensional data to develop a unified unsupervised framework for comparing the profile of more than 4.5 million presynaptic events among normal Hu, NHP, and Mu samples. That is, we used a fully connected neural network^59^ that has proven its effectiveness on large single-event datasets^17,60^. Indeed, this approach provides an effective framework to handle our large and heterogeneous multi-species dataset and perform direct comparisons, while being a suitable solution to simultaneously derive a conjoint cross-species low-dimensional representation and identify differences between the observed presynaptic events groups. We used the autoencoder paradigm, a non-linear embedding method, to derive a compressed low dimensional representation of the input data^61^ while jointly clustering it in an unsupervised way using a loss that preserves the local structure of the input data, critical to perform accurate clustering of the data.

We trained the neural network models with single-event vectors, representing expression across 20 markers, sampled equally from multiple species from the same region. Pooling the data enables direct comparisons between species. Three independent models were trained using samples from different brain regions: cerebral cortex (Hu, NHP, and Mu), hippocampus (Hu and Mu), and neostriatum (Hu and NHP). For each region, a balanced dataset was obtained by downsampling the number of events, stratified by species, to have a similar proportion of events across species and avoid creating a biased integrated space toward one dominating species.

Before applying clustering, we first pretrained the two autoencoders to learn a common low dimensional representation of the multi-species input data by minimizing the standard mean square error (MSE) loss (https://github.com/tpjoe/SynTOF2021)^17^. Then, the weights of the network were fine-tuned minimizing the reconstruction loss as well as a clustering loss^62^, added on top of the low dimensional representation of the two autoencoders. The two autoencoders consist of sequences of fully connected layers with a bottleneck in the middle that imposes a compressed representation of the original input. The optimal number of clusters was derived using the Elbow method and the centers of the clusters were initiated using the K-means algorithm. Both trainings were performed using an adaptive gradient method (Adagrad)^63^, with an initial learning rate of 0.1 and a batch size of 1024. A common Glorot scheme^64^ was adopted to initialize the weights of the networks. The optimal number of epochs was derived automatically exploiting the early stopping algorithm. Finally, the stochastic nature of the training was reduced by repeating the whole training procedure 10 times. Cluster robustness was assessed by applying a consensus meta-clustering which combines clusters from 10 runs using a greedy algorithm^65^, resulting in 15 clusters in the prefrontal cortex (Fscore = 0.776, NMI = 0.702). No hyperparameter selection process was employed. Our model was implemented in Python using Keras^66^. The architecture of the model with its parameterization is represented in Fig. S6.

For dimension reduction, we used the python implementation of tSNE algorithm from scikit learn library^67^, which has a random initialization of the point position. Reproducibility of the figures was ensured by fixing the initialization.

### Clustering validation

We validated the consistency of the generated clustering partition using a silhouette score^32^. This score represents the similarity of an event to events from its own group compared to events from other groups. Comparing silhouette scores based on event clustering defined by model (${s}_{cluster}$), based on species grouping (${s}_{species}$) and subject grouping (${s}_{subject}$), allowed us to quantify bias in learned low dimensional embedding regarding species or subject origin^26^. The silhouette score of overall partitioning generated by the model reached 0.6, whereas the silhouette score obtained by grouping single events based on species origin or subjects was 0.1 or − 0.1, respectively.

To gain further insight into how the model generates the clustering of the single presynaptic events, we leveraged earth mover’s distance (EMD)^68^, which measures the similarity between distributions and quantifies the impact of confounders in CyTOF data^26,31^. Briefly, we computed the pairwise EMD between marker expression distribution of different species across clusters for each marker in cerebral cortex. A significantly lower mean EMD (P-value < 0.001) was found when comparing marker expression between species within the same clusters to different clusters for almost all markers (Fig. S2A–D).

### Statistical analysis

Coefficient of variation (CV) is a popular metric to quantify the homogeneity and the spread of the distribution and compare the relative variability between datasets^69^. For instance, it has been widely used to quantify the batch effect on the data variance^70,71^. Here, CV was used to quantify the effect of pooling multiple species SynTOF data together on the marker expression variance by comparing the CV computed for Hu data only with the CV computed for combined data from multiple species (Fig. 1D). There was no significant difference between mean coefficient of variation (CV) from the two datasets (t-Test P-value = 0.79 in cerebral cortex = 0.99 in neostriatum, and = 0.98 > 0.05 in hippocampus), demonstrating that significant variation in marker distribution spread was not observed when pooling data from different species.

### Graph-based analysis

A Pearson correlation graph was built using the spring layout of the Networkx Python package (https://networkx.org/documentation/stable/), based on the Fruchterman-Reingold algorithm^72^. Edge weights were set equal to the absolute correlation coefficient between nodes. P-values were derived using the Pearsonr function from Python package Scipy^73^ and adjusted for multiple hypothesis testing using Bonferroni correction. Only significant edges above the global mean edge value were drawn. For visualization purposes, we filtered out correlation coefficients lower than the mean correlation coefficient (Figs. 2E, 3B, S4F).

Proximity between subjects across clusters was determined using a nearest-neighbor graph built on single-event mean expression (Fig. 1E). To do so, we set edges equal to the normalized Euclidean distance between the nodes and only significant edges above the global mean edge value were drawn. A similar layout was used for this graph.

### Transcriptomic data (Single nucleus RNA-seq)

Transcriptomic data (single nucleus RNA-seq) from the three species from motor cortex provided by^33^ were used to perform the pseudo-bulk analysis. Statistical analysis was performed in Python using Scipy library^73^ on all SCT normalized neuronal single cells^74^.

### Visualization

Figures have been created using Matplotlib and seaborn packages in Python. Biorender was used to generate subfigures in Figs. 1, 2, 4, S2 and S4.

### Supplementary Information

Supplementary Information.

## Data Availability

The codes are available at https://github.com/elo-nsrb/SynTOF_Cross-species_analysis. The raw SynTOF data are available on Dryad at https://doi.org/10.5061/dryad.z612jm6cr (see^18^ for more information).
