# Supplementary material for: Cross-species comparative analysis of single presynapses
Source: Sci Rep. 2023 Aug 24;13:13849. doi: 10.1038/s41598-023-40683-8 (PMC10449792; doi:10.1038/s41598-023-40683-8)
Supplement: Supplementary file 1 — Supplementary Information. [file 41598_2023_40683_MOESM1_ESM.pdf]

SUPPLEMENTARY MATERIALS

Cross-species comparative analysis of single presynapses

Authors: Eloïse Berson[1,2], Chandresh R. Gajera[1], Thanaphong Phongpreecha[1,2], Amalia Perna[1], Syed A. Bukhari[1], Martin Becker[2], Alan L. Chang[2], Davide De Francesco[2], Camilo Espinosa[2], Neal G. Ravindra[2], Nadia Postupna[3], Caitlin S. Latimer[3], Carol A. Shively[4], Thomas C. Register[4], Suzanne Craft[5], Kathleen S. Montine[1], Edward J. Fox[1], C. Dirk Keene[3], Sean C. Bendall[2], Nima Aghaeepour[2,6,7], Thomas J. Montine[1]

Affiliations:

[1]Department of Pathology, Stanford University; Stanford, CA, United States

[2]Department of Anesthesiology, Perioperative, and Pain Medicine, Stanford University; Stanford, CA, United States

[3]Department of Laboratory Medicine & Pathology, University of Washington; Seattle, WA, United States

[4]Department of Pathology/Comparative Medicine, Wake Forest School of Medicine; Winston-Salem, NC, United States

[5]Department of Internal Medicine–Geriatrics, Wake Forest School of Medicine; Winston-Salem, NC, United States

[6]Department of Pediatrics, Stanford University; Stanford, CA, United States

[7]Department of Biomedical Data Science, Stanford University; Stanford, CA, United States

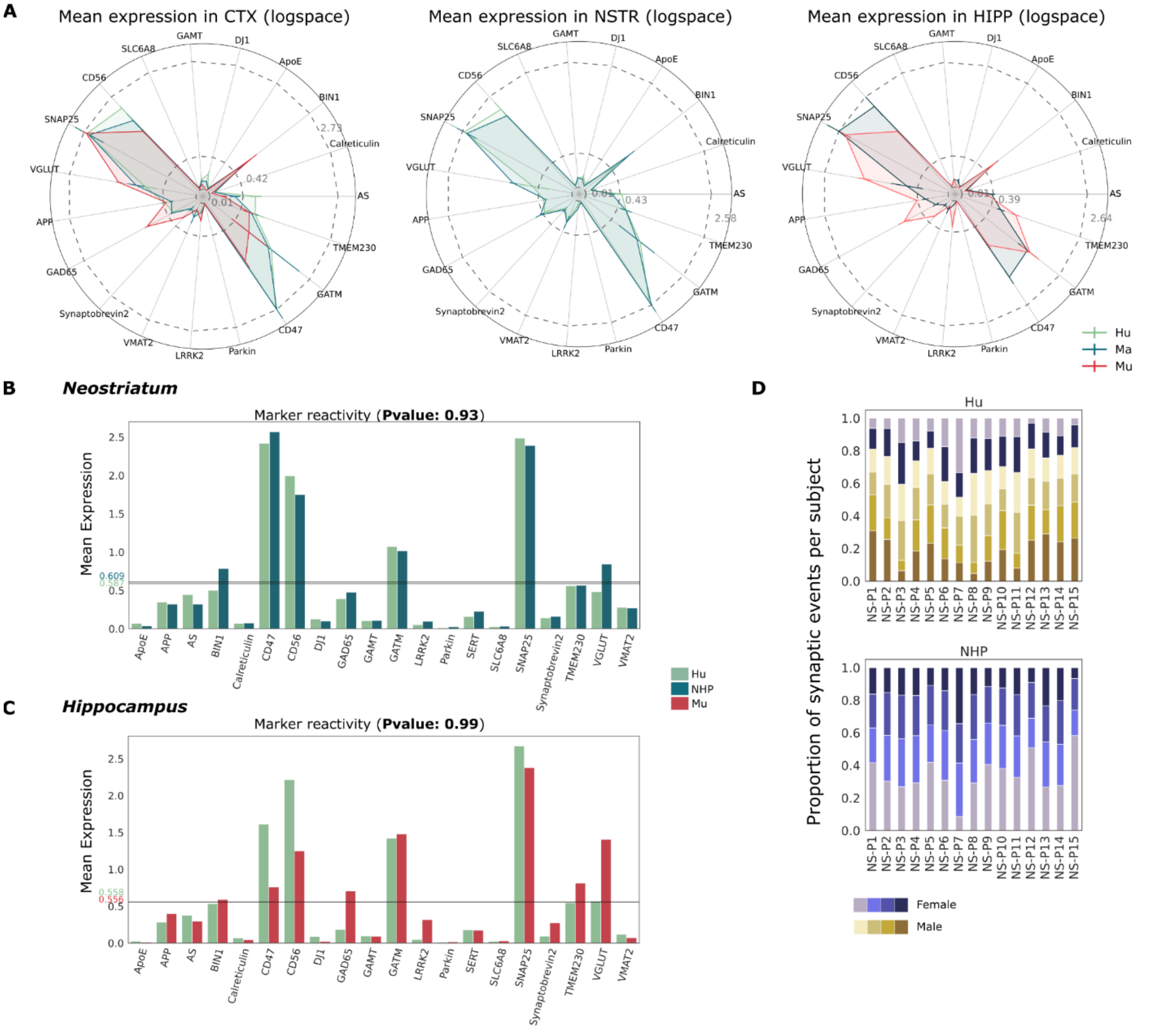

Figure S1. Presynaptic signals across species suggest non-zero marker expression and a minimal impact of marker reactivity in three brain regions.

(A) Non-zero mean marker expressions per species in the cerebral cortex (CTX), Neostriatum (NSTR) and Hippocampus (HIPP). (B) Barplot of mean cross-reactive marker expression levels for Hu and NHP in neostriatum: T-test revealed no significant differences between the two species' mean levels (P-value >> 0.05). (C) Barplot of mean cross-reactive marker expression levels for Hu and Mu in hippocampus. T-test revealed no significant differences between the two species' mean levels (P-value >> 0.05). (D) The proportion of subject-specific synaptic events per cluster stratified by species. Presynaptic events across samples grouped together based on clustering assignment; No clusters were clearly segregated by subjects or sex, suggesting that our method is unaltered by intra-species or sex variation.

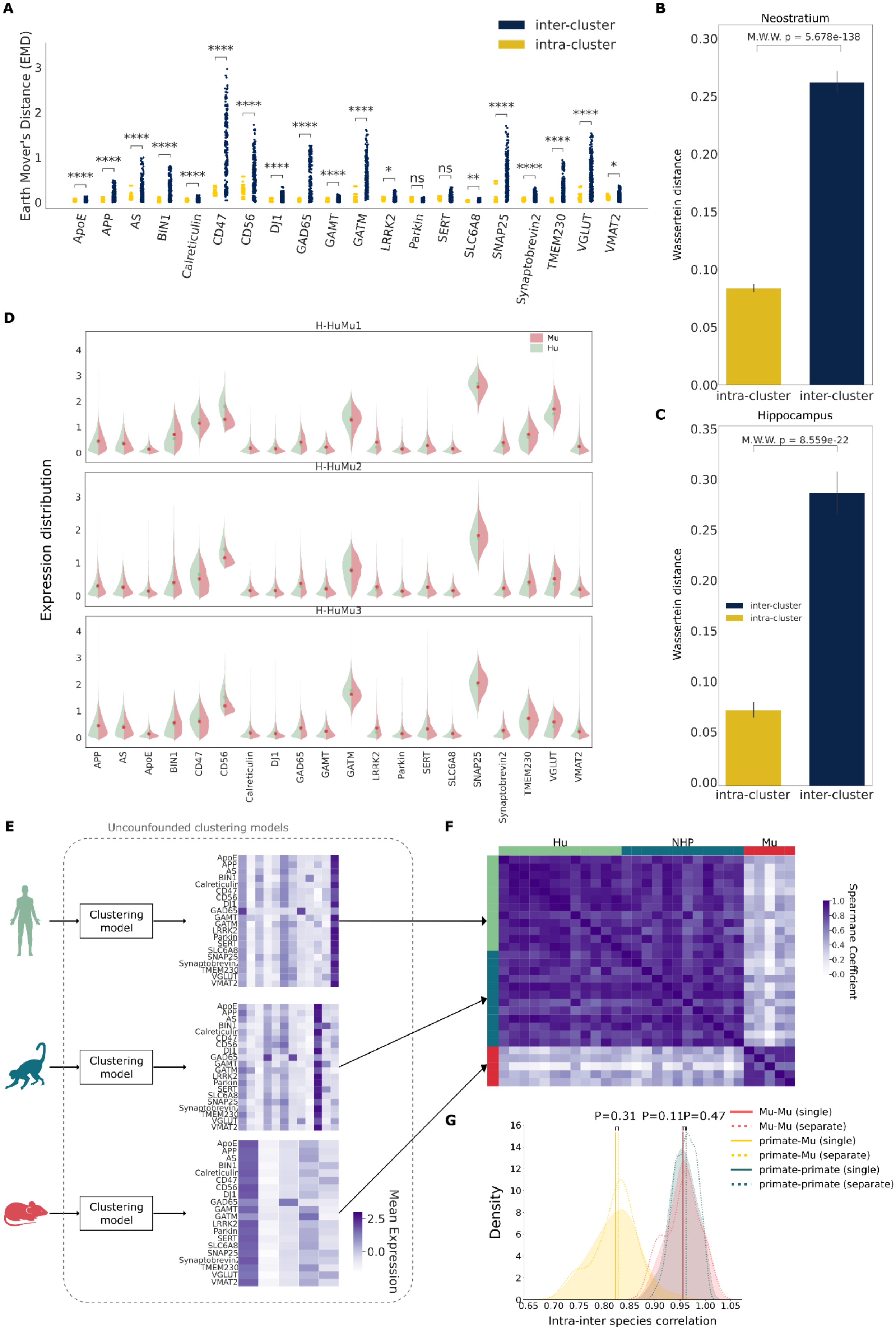
A
inter-cluster
intra-cluster
Earth Mover's Distance (EMD)
ApoE APP AS BIN1 Calreticulin CD47 CD56 DJ1 GAD65 GAMT GATM LRRK2 Parkin SERT SLC6A8 SNAP25 Synaptobrevin2 TMEM230 VGLUT VMAT2
B
Neostratium
M.W.W. p = 5.678e-138
Wassertein distance
intra-cluster
inter-cluster
C
Hippocampus
M.W.W. p = 8.559e-22
Wassertein distance
inter-cluster
intra-cluster
D
H-HuMu1
H-HuMu2
H-HuMu3
Mu
Hu
Expression distribution
APP AS ApoE BIN1 CD47 CD56 Calreticulin DJ1 GAD65 GAMT GATM LRRK2 Parkin SERT SLC6A8 SNAP25 Synaptobrevin2 TMEM230 VGLUT VMAT2
E
Uncounfounded clustering models
Clustering model
Clustering model
Clustering model
Mean Expression
F
Hu
NHP
Mu
Spearmane Coefficient
G
P=0.31 P=0.11P=0.47
Mu-Mu (single)
Mu-Mu (separate)
primate-Mu (single)
primate-Mu (separate)
primate-primate (single)
primate-primate (separate)
Density
Intra-inter species correlation

Figure S2 . Machine learning clustering model minimally impacted by technical confounders.
(A) Pairwise EMD between primate's protein expression from the same (intra-) or different (inter-) clusters per protein in the cerebral cortex. Significant differences (P-value < 0.001(***) after Benjamini-Hochberg corrected Wilcoxon's testing) between the two groups are observed in almost all proteins suggesting that the model aligns single events based on their marker expression distribution. Non-significant differences are annotated as NS. (B&C) Earth mover's distance (EMD) comparison between marker distributions of Hu and NHP samples and Hu and Mu from the same or different clusters in neostriatum (B) and hippocampus (C). A significantly (by t-test) lower EMD mean value was observed when comparing distributions within a cluster than between clusters. Comparisons were performed using multi-species clusters only. (D) Violin plots displaying marker distributions of Hu and Mu in the three common clusters H-HuMu1-3 in hippocampus. Cross-species protein-level distributions were more consistent within clusters than across clusters, even when clustering highly heterogeneous multi-species datasets. Despite differences in protein levels between Hu and Mu in the hippocampus, the algorithm grouped together events from Hu and Mu that shared similar patterns in presynaptic protein expression. (E-G) Model validation: (E) First, we clustered the events from the three species independently, leading to clusters that are unaffected by antibody avidity. (F) Then, we compared species-specific clusters by computing the similarity between the mean cluster expression. (G) Finally, statistical analysis revealed no significant difference between the mean intra/inter species correlation between the presynaptic population of primates (Hu and NHP) and NHP across clusters derived using either single common model for all species (single) or a separate model for each species (separate) from corrected Wilcoxon's test.

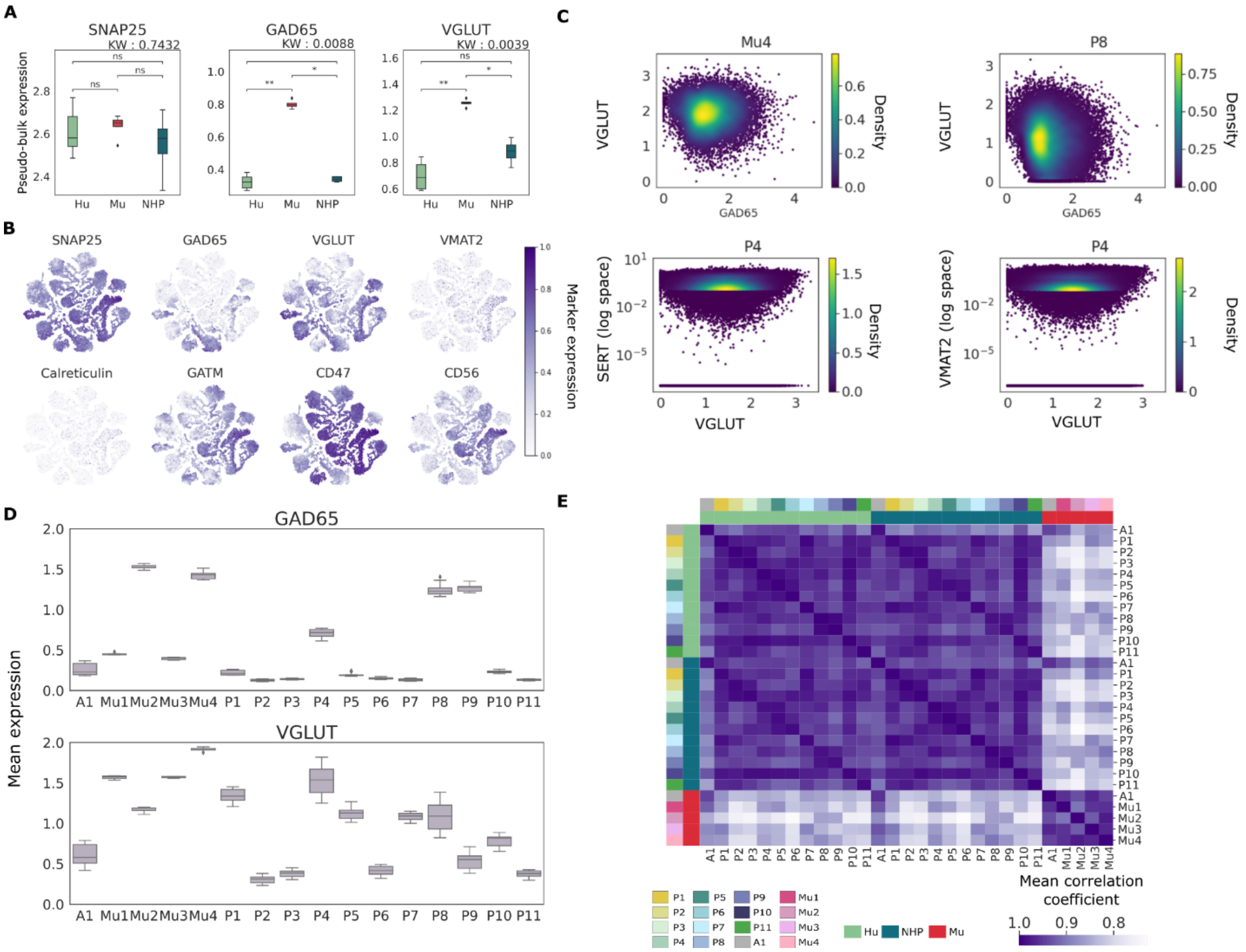

Figure S3. Single-synapse comparison in cerebral cortex across the three species.
(A) Pseudo-bulk differential expression of SNAP25, GAD65 and VGLUT in cerebral cortex between three species. While no significant difference was observed in SNAP25 abundance across species (Kruskal-

Wallis P-value >0.05), a significantly higher expression of VGLUT and GAD65 was observed in Mu compared with primates. (B) Original t-SNE overlaid with marker abundance profiles. The colormap shows marker expression values after logarithm transformation and minMax normalization. (C) Biplot of GAD65 and VGLUT co-expression in Mu4 and P8, and VMAT2, VGLUT and SERT in P4. (D) Box plot of GAD65 and VGLUT mean marker expression per cluster in cerebral cortex. (E) Interspecies correlation matrix of mean expression vector per clusters in cerebral cortex after projection in the autoencoder latent space. The evolutionary tree is observed at mean expression level. Overall, correlation coefficients were higher between Hu and NHP than between Hu and Mu, suggesting a stronger proximity between mean expression within primate groups than between primate-Mu interspecies groups.

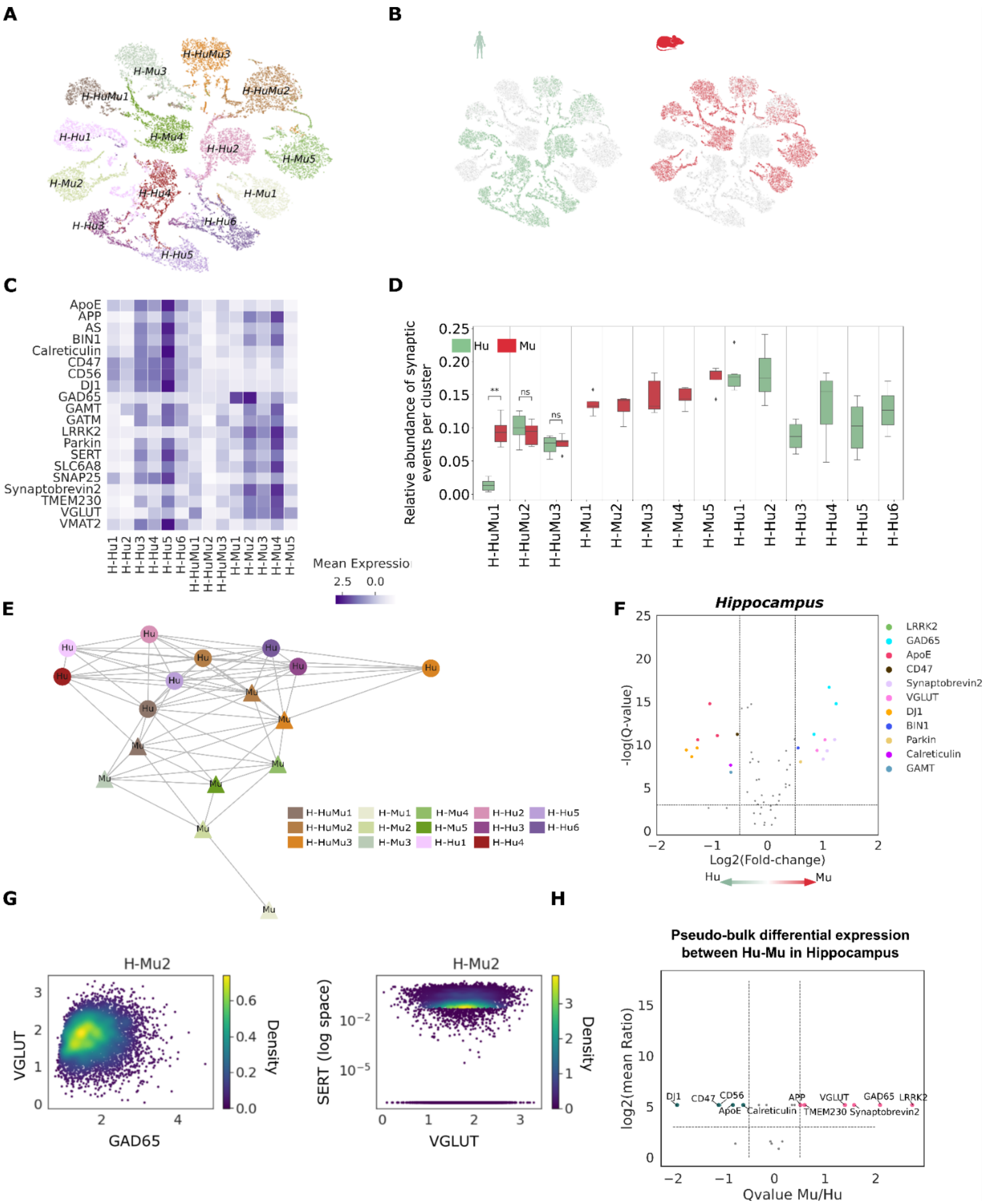

Figure S4. Comparative analysis between Hu and Mu presynaptic events in hippocampus.
(A) t-SNE of presynaptic events after nonlinear dimension reduction colored by clusters. (B) t-SNE of presynaptic events after nonlinear dimension reduction colored by species. (C) Row-normalized cross-species mean expression heatmap of 20 markers per cluster. (D) Mean frequency of synaptic events per cluster afterS removing events present in less than 0.01. Symbols indicate significant differences using Wilcoxon's P-value <0.05 (*) after Benjamini-Hochberg. (E) Graph based on the Fruchterman-Reingold

algorithm of mean expression values displaying the underlying organization of hippocampal clusters. Nodes represent mean expression vectors embedded in latent space, while edges indicate Pearson correlation coefficients after Bonferroni correction (P-value >0.05). Only edges superior to the mean Pearson correlation value were drawn. (F) Volcano plot of differential marker expression between Hu and Mu in hippocampus. Only significantly different marker expressions after multiple testing corrected Wilcoxon's test are colored. (G) VGLUT, GAD65 and SERT co-expression in H-Mu2. (H) Volcano plot of the pseudo-bulk differential protein expression between Hu and Mu in hippocampus (as noted in Figure 1A, NHP hippocampus was not available) after multiple testing corrected Wilcoxon's Test using Benjamini-Hochberg method. Only significantly different marker expressions are colored.

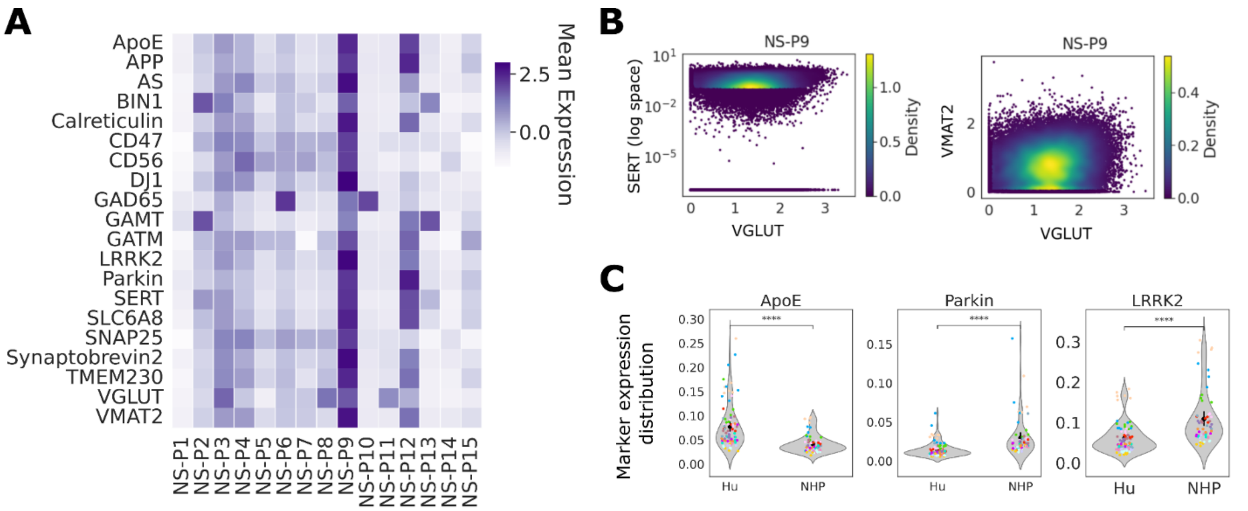

Figure S5. Comparative analysis between Hu and NHP presynaptic events in neostriatum
(A) Row-normalized cross-species mean expression heatmap of 20 markers per cluster. (B) VGLUT, VMAT2 and SERT co-expression in NS-P9. (C) Violin plot showing markers' distribution of significantly different mean protein expression between the two species per cluster after multiple testing corrected Wilcoxon's test in neostriatum.

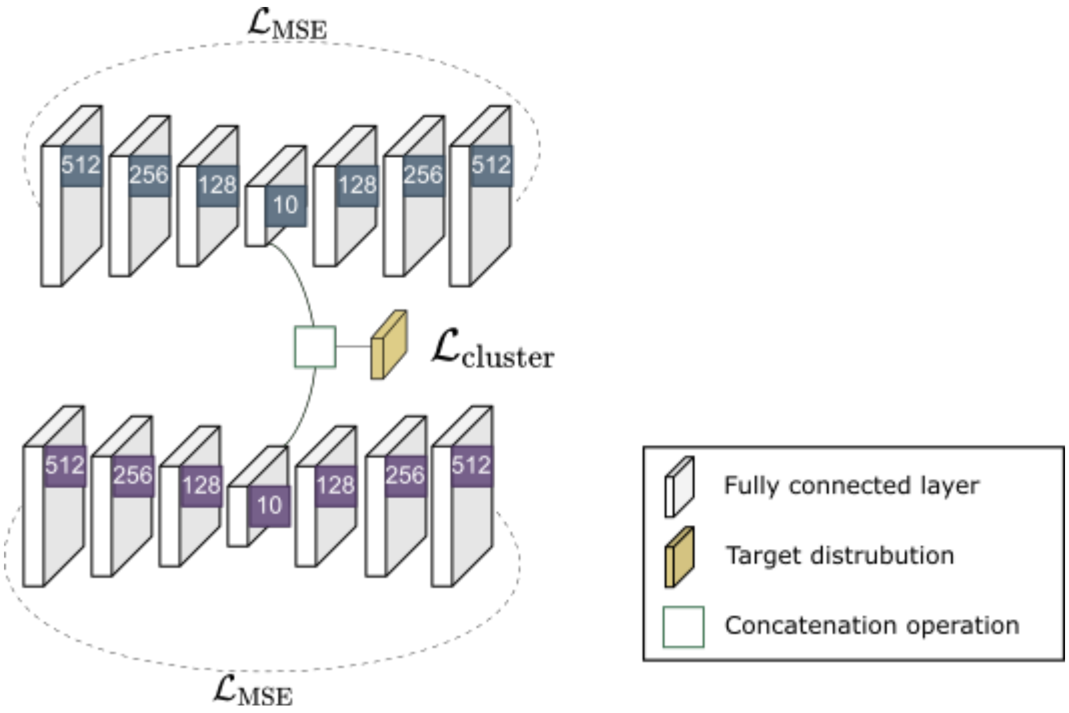

Figure S6. Neural network architecture.
Based on two autoencoders, the neural network jointly learns a low dimensional meaningful representation of the input data and clusters the data in an unsupervised way. This is achieved by first training the two autoencoders separately, optimizing the reconstruction loss (Mean Square Error), and then simultaneously fine-tuning both networks by conjointly optimizing the reconstruction and clustering losses proposed by Xie and colleagues [1].

SUPPLEMENTARY METHODS

Overview: All SynTOF data used in the present study were generated in a previous publication[2] and are publicly available. As described therein, human, macaque, and mouse synaptosome were prepared using established protocols[3] modified for CyTOF analysis[4,5] including mass tag barcoding[6]. All protocols were approved by the Institutional Review Board or Institutional Animal Care and Use Committee. Each synaptosome preparation yielded barcode-assigned, sequentially gated events, with each event defined using a 38-antibody panel of validated antibodies to exclude non-synaptic particles and to characterize individual presynaptic events. Technical variability was minimized by using the same master mix antibody cocktail, and each sample was barcoded and combined to pool sample acquisition, thereby minimizing cross sample variability[6]. In addition, acquisitions were done at the same site using the same mass cytometer with exactly the same parameters and settings for all samples across species. Previous quality control analysis showed that Hu synaptosomes are normally distributed about a mean of approximately 8 fL[4], and that NHP and Mu synaptosomes are broadly overlapping in size without significant difference from Hu[3,7].

Presynapse data: The SynTOF panel comprised all available phenotypic markers (20/24) from the publicly available data : the other four (PrP, Tau, GBA1 and DAT) were excluded due to lack of validated cross-reactivity (as noted in[2]). Analysis focused on presynaptic data as they make up ~90% of events in synaptosome preparations. Four original panel antibodies (CD11b, gephyrin, PSD95, and MBP) were used for manual gating to exclude nonpresynaptic events.

Previously described optimization and validation steps: The components of SynTOF[3–8] were developed to ensure technical rigor at each step. Indeed, the initial three SynTOF publications detail the its extensive optimization to detect single human synaptic events with preserved molecular integrity in synaptosome preparations free of homogenate debris or doublets/multiplets that contaminate even the most stringent synaptosome preparations not subjected to quality control as described below.

<u>Synaptosome samples</u>. Several labs have adapted conventional flow cytometric analysis to synaptosomes from human, mouse, and NHP[3,7–11]. Despite using different samples, probes, and instruments, synaptosome cytometry is a robust and reproducible technique across labs. Synaptosomes preparation methods have been refined over many years across multiple labs, and include homogenization under strict conditions followed by filtration[4,12,13]. Highly effective cryopreservation (500 µl aliquots in 10% DMSO in FBS) has been confirmed for once-thawed preparations[6].

<u>Barcoding</u>. Mass-tag cell barcoding is widely used in cytometry[14–16], reducing intra-sample variation, since up to 20 samples can be barcoded, mixed, and then incubated with a single antibody cocktail. The adaptation of Dr. Eil Zunder's protocol[17] to synaptosomes[6] reduced errors arising from differences in preparations or variability across samples. In addition, because the debarcoding step removes particles with conflicting barcode patterns, it minimizes debris inherent in synaptosome preps and doublets/multiplets arising from the small event size.

Antibody authentication. Enhanced antibody authentication standards proposed by the International Working Group for Antibody Validation[18] are used for all SynTOF studies. Antibodies have either genetic validation, the first level of authentication, or orthogonal validation, the second level, as used by the Human Protein Atlas (HPA)[18,19]. Antibodies are routinely characterized to ensure they meet necessary standards for the proposed research. For each clone and batch, they are authenticated by Western blot and immunofluorescence using knockout cells, positive and negative controls, as well as species controls as available. Overall, 166 antibodies that met these criteria have been screened; 38 were brought forward for the previously published study [1] (shown in Table S4). Additional antibody QC steps include: (i) only conjugated antibodies with Western blot banding pattern expected from the literature and unchanged from non-conjugated antibody are included; (ii) metal minus controls, where synaptosomes are incubated without conjugated antibody to determine background signal in the omitted channel; (iii) isotype antibodies obtained from the same supplier as the primary antibody whenever possible, always from the same host species and Ig subclass, conjugated with the same metal ion and used at the same concentration as the primary antibody conjugate. Signal overlapping with isotype control is excluded as non-specific[5].

Molecular Integrity of Human Synaptosomes. Additional rigor built into synaptosome method development includes demonstrating the structural and molecular integrity of human synaptosomes collected following death. Indeed, despite all of human samples coming from individuals who were carefully followed as part of research cohorts, it is not possible to completely control for the potential impact of chronic systemic illnesses, agonal state, and short post-mortem interval of samples obtained from humans. The potential impact of these variables has been accounted for by comparing human and NHP synaptosome data, i.e., monkeys culled to control colony size (no preceding illness) and euthanized under deep anesthesia by perfusion with ice-cold saline (no agonal state or post-mortem interval).

Quality Control for Single-Synapse Data. Unlike cell suspensions that are used in conventional flow cytometry and CyTOF, synaptosomes derive from brain homogenates and thus contain variable amounts of debris depending on the method of preparation. The first quality control step is to use the most stringent method of synaptosome preparation. The second quality control step applies six sequential exclusion or inclusion gates while still retaining a large number of mass channels for data collection[4,6]. Having eliminated even minute debris, the third quality control step is mass-tag barcoding[6]. Applying these multiple quality checks eliminates ~85% of the total events in the original synaptosome preparation, and yields highly enriched single pre- and postsynapses[2,6]. Such robust quality control distinguishes this approach to single-synapse analysis from other methods currently in use.

Validation. Three methods have been used to validate SynTOF results. External validation can be conducted by comparing average results with bulk analyses done by others, and by comparing single synapse results with array tomography data. Internal validation by MIBI uses the same type of conjugated antibodies as SynTOF, but collects data in situ from fixed tissue. MIBI on human brain has been used to validate major SynTOF findings concerning increased CD47 expression in situ, while controlling for the possibility that tissue homogenization somehow corrupted the results[2].

SUPPLEMENTARY METHODS REFERENCES

1. Xie, J., Girshick, R. & Farhadi, A. Unsupervised Deep Embedding for Clustering Analysis. in *Proceedings of the 33rd International Conference on Machine Learning* (eds. Balcan, M. F. & Weinberger, K. Q.) vol. 48 478–487 (PMLR, 20--22 Jun 2016).
2. Phongpreecha, T. *et al.* Single-synapse analyses of Alzheimer's disease implicate pathologic tau, DJ1, CD47, and ApoE. *Sci Adv* **7**, eabk0473 (2021).
3. Postupna, N. O. *et al.* Flow cytometry analysis of synaptosomes from post-mortem human brain reveals changes specific to Lewy body and Alzheimer's disease. *Lab. Invest.* **94**, 1161–1172 (2014).
4. Gajera, C. R. *et al.* Mass synaptometry: High-dimensional multi parametric assay for single synapses. *J. Neurosci. Methods* **312**, 73–83 (2019).
5. Gajera, C. R. *et al.* Mass Synaptometry: Applying Mass Cytometry to Single Synapse Analysis. *Methods Mol. Biol.* **2417**, 69–88 (2022).
6. Gajera, C. R. *et al.* Mass-tag barcoding for multiplexed analysis of human synaptosomes and other anuclear events. *Cytometry A* **99**, 939–945 (2021).
7. Postupna, N. *et al.* Human Striatal Dopaminergic and Regional Serotonergic Synaptic Degeneration with Lewy Body Disease and Inheritance of APOE ε4. *Am. J. Pathol.* **187**, 884–895 (2017).
8. Postupna, N. O. *et al.* Flow cytometric evaluation of crude synaptosome preparation as a way to study synaptic alteration in neurodegenerative diseases. *Neuromethods* **141**, 297–310 (2018).
9. Bilousova, T. *et al.* Synaptic Amyloid-β Oligomers Precede p-Tau and Differentiate High Pathology Control Cases. *Am. J. Pathol.* **186**, 185–198 (2016).
10. Gylys, K. H. & Bilousova, T. Flow Cytometry Analysis and Quantitative Characterization of Tau in Synaptosomes from Alzheimer's Disease Brains. *Methods Mol. Biol.* **1523**, 273–284 (2017).

11. Sokolow, S. *et al.* Isolation of synaptic terminals from Alzheimer's disease cortex. *Cytometry A* **81**, 248–254 (2012).

12. Hollingsworth, E. B. *et al.* Biochemical characterization of a filtered synaptoneurosome preparation from guinea pig cerebral cortex: cyclic adenosine 3':5'-monophosphate-generating systems, receptors, and enzymes. *J. Neurosci.* **5**, 2240–2253 (1985).

13. Koffie, R. M. *et al.* Apolipoprotein E4 effects in Alzheimer's disease are mediated by synaptotoxic oligomeric amyloid-β. *Brain* **135**, 2155–2168 (2012).

14. Hartmann, F. J., Simonds, E. F. & Bendall, S. C. A Universal Live Cell Barcoding-Platform for Multiplexed Human Single Cell Analysis. *Sci. Rep.* **8**, 10770 (2018).

15. Mei, H. E., Leipold, M. D., Schulz, A. R., Chester, C. & Maecker, H. T. Barcoding of live human peripheral blood mononuclear cells for multiplexed mass cytometry. *J. Immunol.* **194**, 2022–2031 (2015).

16. Rybakowska, P., Alarcón-Riquelme, M. E. & Marañón, C. Key steps and methods in the experimental design and data analysis of highly multi-parametric flow and mass cytometry. *Comput. Struct. Biotechnol. J.* **18**, 874–886 (2020).

17. Zunder, E. R. *et al.* Palladium-based mass tag cell barcoding with a doublet-filtering scheme and single-cell deconvolution algorithm. *Nat. Protoc.* **10**, 316–333 (2015).

18. Barderas, R., LaBaer, J. & Srivastava, S. *Protein Microarrays for Disease Analysis: Methods and Protocols*. (2021).

19. Zelanis, A. *Proteolytic Signaling in Health and Disease*. (Academic Press, 2021).
